# Plant growth–promoting traits of culturable seed microbiome of citrus species from Purvanchal Himalaya

**DOI:** 10.3389/fpls.2023.1104927

**Published:** 2023-07-10

**Authors:** Sakshi Sinha, Dwipendra Thakuria, Chayanika Chaliha, Panchali Uzir, Samarendra Hazarika, Pranab Dutta, A. K. Singh, Bingiala Laloo

**Affiliations:** ^1^School of Natural Resource Management, College of Post Graduate Studies in Agricultural Sciences, Central Agricultural University (Imphal), Umiam, Meghalaya, India; ^2^Division of System Research and Engineering, Indian Council of Agricultural Research (ICAR) Research Complex for North Eastern Hill Region, Umiam, Meghalaya, India; ^3^School of Crop Protection, College of Post Graduate Studies in Agricultural Sciences, Central Agricultural University (Imphal), Umiam, Meghalaya, India; ^4^School of Crop Improvement, College of Post Graduate Studies in Agricultural Sciences, Central Agricultural University (Imphal), Umiam, Meghalaya, India

**Keywords:** phosphate complexes dissolution, zinc complexes dissolution, IAA production, N2 fixation, siderophore, microbial consortium

## Abstract

Despite Northeastern India being “Treasure House of Citrus Genetic Wealth,” genetic erosion of citrus diversity poses severe concern with a corresponding loss in seed microbial diversity. The seed microbiome of citrus species unique to the Purvanchal Himalaya is seldom explored for their use in sustainable orchard management. Isolation and characterization of culturable seed microbiomes of eight citrus species, namely, *Citrus reticulata* Blanco, *C. grandis* (L.) Osbeck, *C. latipes* Tanaka, *C. megaloxycarpa* Lushaigton, *C. jambhiri* Lush, *C. sinensis* (L.) Osbeck, *C. macroptera* Montr, and *C. indica* Tanaka collected from NE India were carried out. The isolates were then screened for an array of plant growth–promoting (PGP) traits [indole acetic acid (IAA) production, N_2_ fixation, phosphate and zinc complex dissolution, siderophores, and Hydrogen Cyanide (HCN) production]. The pure culture isolates of seed microbiomes were capable of dissolving insoluble Ca_3_(PO_4_)_2_ (1.31–4.84 µg Pi ml^-1^ h^-1^), Zn_3_(PO_4_)_2_ (2.44–3.16 µg Pi ml^-1^ h^-1^), AlPO_4_ (1.74–3.61 µg Pi ml^-1^ h^-1^), and FePO_4_ (1.54–4.61µg Pi ml^-1^ h^-1^), mineralized phytate (12.17–18.00 µg Pi ml^-1^ h^-1^) and produced IAA-like substances (4.8–187.29 µg ml^-1^ h^-1^). A few isolates of the seed microbiome were also able to fix nitrogen, secrete siderophore-like compounds and HCN, and dissolve ZnSO_4_ and ZnO. The 16S ribosomal Ribonucleic Acid (rRNA)–based taxonomic findings revealed that *Bacillus* was the most dominant genus among the isolates across citrus species. Isolates CG2-1, CME6-1, CME6-4, CME6-5, CME6-9, CJ7-1, CMA10-1, CI11-3, and CI11-4 were identified as promising bioinoculants for development of microbial consortium having multifaceted PGP traits for nutritional benefits of nitrogen, phosphorus and zinc, and IAA hormonal benefits to citrus crops for better fitness in acid soils.

## Introduction

Plants, as metaorganisms, are associated with diverse microbes from various niches (such as the rhizosphere, spermosphere, phyllosphere, and endosphere). The interaction between plant and microbes may be either casual or intimate, but they all furnish an evolving seed microbiome that may carry over to other developmental stages found often in plants growing in natural situations (Hardoim et al., 2015). Bacterial endophytes are all bacteria that may or may not be effectively cultured and can colonize a plant internally without causing apparent harm or creating any obvious exterior structures (Azevedo and Araujo, 2007). Endophytes with plant growth–promoting (PGP) traits are generally the most effective in promoting growth because they live inside plant tissues and can communicate with the host plant and exert their beneficial effect much more effectively (Giauque et al., 2019). Protected from the outside environment, endophytes are much less susceptible to the soil’s frequent chemical–physical biotic and abiotic variations. The beneficial action of these bacteria is primarily expressed by directly promoting nutrient absorption via modulation of plant hormone levels (Marchut-Mikolajczyk et al., 2018). Seed-borne endophytes lay the foundation for the development of plant microbiomes as the pioneer colonizers, and seeds serve as carriers for the advantageous microorganisms for multigenerational preservation of these beneficial relationships (Truyens et al., 2015). Association of seed and endophytes have been documented in a variety of crops including rice (Cottyn et al., 2001), maize (Rijavec et al., 2007), bean (López-López et al., 2010), wheat (Kuźniar et al., 2020), and tomato (Xu et al., 2014). The collection of factors that go into the construction and organization of seed microbiomes is quite complex, with microbes drawn from not only the soil microbiome (Hardoim et al., 2012) but also dispersal agents, pollinator, and floral microbiomes (Gandolfi et al., 2013). Only specialized endophytic strains are able to colonize and flourish in reproductive plant organs (Truyens et al., 2015), and particularly, bacteria with competitive and adaptive colonization traits could dwell in seeds (Okunishi et al., 2005). Through a variety of processes, such as the solubilization of phosphate (Berg, 2009), synthesis of phytohormones such as indole-3-acetic acid (Defez et al., 2017), production of 1-aminocyclopropane deaminase (Glick, 2005), nitrogen fixation (Shabanamol et al., 2018), siderophore generation, and inhibition of phytopathogenic microbes (Hong and Park, 2016), seed bacterial endophytes have been reported to enhance plant development and health. Across a wide range of plant taxa including wheat, *Brassica*, rice, and maize, seed-associated bacteria have been identified using next-generation sequencing (NGS) techniques and are found largely within the bacterial phyla Acidobacteria, Proteobacteria, Actinobacteria, Firmicutes, Bacteroidetes, and Deinococcus-Thermus (Johnston-Monje and Raizada, 2011; Liu et al., 2012) with Gammaproteobacteria (Hardoim et al., 2015; Herrera et al., 2016) found to be the most prevalent type of bacteria at the class level, followed by Alphaproteobacteria (Hardoim et al., 2015) and Bacilli (Comby et al., 2009). Saving seeds with their indigenous microbes offers an enormous potential to safeguard, explore, and exploit microbial diversity and their untapped metabolic potential for plant, human, and environmental health.

The citrus fruit is a well-known source of biocompounds crucial for human nutrition and among one of the most commercially significant evergreen subtropical fruit crops in the world (Penyalver et al., 2022). North-eastern India is recognized as a significant hotspot of citrus biodiversity renowned as the “Treasure House of Citrus Genetic Wealth” attributable to the region’s special blend of different soil, physiographic, and climatic conditions (Malik et al., 2013). Domestication of wild plant species has been a historical process that has lasted since the dawn of agriculture. In order to meet changing human demands, wild species are domesticated and diversification of the seed microbiome occurs as a consequence of domestication (Abdullaeva et al., 2021). The northeastern region of the country harbors several wild, semiwild, and domesticated species including *Citrus indica*, *C. jambhiri*, *C. grandis*, *C. macroptera*, *C. latipes*, and *C. megaloxycarpa* aside from the commercially cultivated species like *Citrus reticulata* (Khasi mandarin) and *C. sinenis*. The rapid destruction of forests in the northeastern region has endangered these naturally occurring citrus species and the microbiota associated with them. These species may have great potential in the improvement of Indian Citrus industry by being a source of genes for combating biotic and abiotic stresses like pests and disease resistance, especially resistance to greening disease in its natural habitat (Yang and Ancona, 2022; Jing et al., 2023), tolerance to psorosis and exocortis virus (Sharma et al., 2004), cold tolerance or resistance (Hazarika, 2012), and drought resistance (Pandey et al., 2008). In citrus, the seed-to-seedling phase is very crucial as the seedling is commonly used as rootstock onto which scion varieties are grafted. Numerous ethnomedic benefits of citrus species, which have been used by the local population for centuries, have been discovered. Traditional medicines frequently employ the peel of the *C. grandis* fruit to treat epilepsy, edema, and cough (Anmol et al., 2021); fresh fruit, juice, and dried powder from *C. indica* are used for curing viral diseases, jaundice, and deadly communicable diseases like small pox (Malik et al., 2006). *C. macroptera* fruits are used medicinally to alleviate digestive disorders and stomach pain (Malik et al., 2013), whereas *C. latipes* fruits are used to treat viral infections, digestive problems, and jaundice (Malik et al., 2013). In a recent study, microbial profiling was carried out for five different biocompartments, *viz.* bulk soil, root endosphere, flush, rhizosphere, and flower) of citrus from a single California orchard (Xi et al., 2023). However, except for the work by Newcombe et al. (2018) reporting the presence of one cultivable endophyte per seed in the Rutaceae plant family and the study highlighting *Cutibacterium* and *Acinetobacter* as the most prevalent bacterial genera in seeds and shoots of *C. limon* (Faddetta et al., 2021), seed microbiota of citrus species remain largely unexplored. The present study aimed to explore the culturable members of the seed microbiome of *C. reticulata* Blanco, *C. grandis* (L.) Osbeck, *C. latipes* Tanaka, *C. megaloxycarpa* Lushaigton, *C. jambhiri* Lush, *C. sinensis* (L.) Osbeck, *C. macroptera* Montr, and *C. indica* Tanaka collected from the Purvanchal Himalaya region, carried out 16S rRNA–based taxonomic identification, and screened them for multifaceted PGP traits.

## Materials and methods

### Sampling of fruits

Sampling of citrus fruits from different locations of North East region of India including the Nokrek Biosphere Reserve in the West Garo Hills district of Meghalaya was carried out during October–November, 2021. Approximately 8–10 disease/disorder-free fruits per plant and three plants per species were collected at the physiologically ripen stage (total soluble sugars considered as an indicator; **Table 1**). Samples were temporarily stored at 4°C for a few hours and then at −20°C until further processing. The citrus species along with their assigned names and details are listed in **Table 1**.

**Table 1:** Selected citrus species with their assigned name and obtained microbial isolates.

Citrus species	Scientific name	Assign Name	District	Latitude	Longitude	Altitude	Microbial isolates	TSS
Sikkim orange	*C. reticulata* Blanco	CR1	South Sikkim, Sikkim	27.28 ° N	88.39 ° E	650 m	CR1-1,CR1-2	11.18
Pummelo	*C. grandis* (L.) Osbeck	CG2	West Garo hills, Meghalaya	25.57 ° N	90.29 ° E	423 m	CG2-1,CG2-2	13.80
Khasi papeda	*C. latipes* Tanaka	CL5	East Khasi hills, Meghalaya	25.30 ° N	91.96 ° E	1,668 m	CL5-1,CL5-2,CL5-3, CL5-4,CL5-5	8.50
Sour pummelo	*C. megaloxycarpa* Lushaigton	CME6	South Garo hills, Meghalaya	25.28 ° N	90.47 ° E	80 m	CME6-1,CME6-2, CME6-3,CME6-4, CME6-5,CME6-6, CME6-7,CME6-8, CME6-9	6.50
Rough lemon	*C. jambhiri* Lush	CJ7	West Garo hills, Meghalaya	25.63 ° N	90.34 ° E	632 m	CJ7-1,CJ7-2	8.94
Sweet orange	*C. sinensis* (L.) Osbeck	CS8	West Garo hills, Meghalaya	25.65 ° N	90.35 ° E	589 m	CS8-1,CS8-2,CS8-3	
Khasi mandarin	*C. reticulata* Blanco	CR9	West Garo hills, Meghalaya	25.55 ° N	90.33 ° E	761 m	CR9-1,CR9-2,CR9-3, CR9-4,CR9-5	10.80
Melamesian papeda	*C. macroptera* Montr	CMA10	North Garo hills, Meghalaya	25.84 ° N	90.45 ° E	40 m	CMA10-1,CMA10-2, CMA10-3,CMA10-4	10.00
Indian wild orange	*C. indica* Tanaka	CI11	West Garo hills, Meghalaya	25.52 ° N	90.33 ° E	829 m	CI11-1,CI11-2,CI11-3, CI11-4	8.00

### Extraction of seeds

The fruits were first washed with disinfectant liquid and rinsed with water, followed by sterilization with 70% ethanol under sterile conditions and left to dry. A total of 15 fresh seeds were extracted from each fruit under aseptic conditions using autoclaved tools, which were rinsed with sterilized distilled water for 7–10 times (based on differences in the fibers and flesh coating of the seeds) until the surrounding fibers and mucilage were removed. The extracted seeds were dried and stored in sterile falcon tubes along with the fruit juice at 4°C until use.

### Isolation and purification of bacterial microbiota from seeds

#### Surface sterilization of seeds

Three seeds per citrus species were surface-sterilized, which consisted of the following steps: (1) agitation of the seeds in sodium hypochlorite solution (2.0%) for 2 min, which was repeated twice and (2) immersion in 70% (v/v) ethanol for 1 min, followed by repeatedly rinsing the seeds with autoclaved distilled water. To confirm that the sterilization process was successful, 1 ml aliquot of the last-step washing water was plated on growth agar-media and examined for microbial growth after incubation at 30°C for 3–5 days.

#### Cultivation of microbiota from the seeds

The surface-sterilized seeds were activated before by incubating them with adequate amount of sterile water overnight at 28°C. Once seed surface sterility was confirmed, three seeds per species were ground gently in an autoclaved mortar using 0.50 ml of sterilized distilled water and the ground seed suspension (100 μl) was plated on nutrient agar media plates followed by incubation for 2–3 days at 28 ± 0.5°C. The colonies obtained were selected on the basis of colony morphology and/or pigmentation and repeatedly streaked on fresh nutrient agar-media to obtain pure cultures. The purified cultures were maintained in nutrient agar slants and stored at 4°C. The pure isolates were finally cultured in Luria-Bertani (LB) broth for glycerol stocks preparation and stored at -20°C.

### Morphological characterization

The microorganisms isolated from citrus seed were examined for colony size, shape of colony, color of colony, surface of colony, margin, and elevation. The isolated microbial cultures were streaked on nutrient agar plate, incubated at 28 ± 0.5°C, and observed for colony morphology after 48 h.

### *In vitro* test for multiple plant growth–promoting traits

#### Production of indole acetic acid–like substances

An aliquot of 0.1 ml suspension of the pure cultures incubated for 24 h at 30 ± 0.5°C was inoculated in nutrient broth containing 1% L-tryptophan and incubated at 30°C for 48 h at 150 rpm followed by determination of IAA in a supernatant. Pink color was developed by the adding van Urk Salkowski reagent, and the intensity is measured in a spectrophotometer (Spectrascan 2600, Thermo Scientific, USA) at 530 nm wavelength. IAA was expressed in μg ml^-1^ h^-1^.

#### N_2_-fixation test

Nitrogen-free bromothymol blue semi-solid agar was inoculated with the pure isolate broth and incubated along with an uninoculated control at 30°C for 7 days. The color of the media was light green in the start, but due to increase in pH of the medium on fixation of N_2_ by the bacterial isolate, the media color turns to blue confirming the capacity of the microorganism to fix N_2_ as described by Okon (1985).

#### Phosphate-solubilization ability test

The ability of pure culture isolates to solubilize insoluble forms of phosphates was determined on the Pikovskaya’s agar plates amended with Ca_3_(PO_4_)_2_, AlPO_4_, FePO_4,_ Zn_3_(PO_4_)_2_, and Na-phytate separately spot-inoculated with culture isolates. Transparent zone formation in the periphery of the individual bacterial colony after 72 h of incubation at 30 ± 0.5°C confirmed the solubilization ability (Pikovskaya, 1948). The quantitative estimation was carried out on Pikovskaya’s broth amended with Ca_3_(PO_4_)_2_, AlPO_4_, FePO_4,_ Zn_3_(PO_4_)_2_, and Na-phytate separately, inoculated with 10 μl of bacteria pure culture. In brief, 100 μl of clear supernatant was taken and 5 ml 0.03N NH_4_F in 0.025 N HCl was added. Development of blue color was carried out using Dickman–Bray’s reagent and stannous chloride. Finally, the intensity of blue color was measured at 660 nm (Spectrasca UV- 2600, Thermo Scientific, USA) and concentration of P was read from the P standard curve (0, 0.08, 0.2, 0.4, 0.6, 0.8, and 1.0 ppm). The concentration of P was expressed as μg ml^-1^ h^-1^.

### Dissolution of insoluble zinc complexes

The zinc-solubilizing efficiency of the pure culture isolates was estimated by the plate assay using modified Bunt and Rovira media–amended 0.1% ZnO and 0.1% ZnSO_4_ separately as a source of insoluble inorganic zinc with bromophenol blue as an indicator. The isolates were spot-inoculated on the plates, and after 48 h of incubation at 30°C, the plates showing a halo zone around the colonies were considered positive for solubilization of inorganic zinc. The diameter of the clear zone was measured to calculate the solubilizing efficiency (SE) (Ramesh et al., 2014).


$$\text{SE}=\frac{\text{Solubilization diameter}-\text{Colony diameter}}{\text{Colony diameter}}\;\times 100$$

### Siderophore production

Universal Chrome Azurol S (CAS) agar medium was prepared as described by Schwyn and Neilands (1987) to check siderophore-producing ability of bacterial isolates. The bacterial strains were spot-inoculated on each plate with an uninoculated plate taken as control. After inoculation, plates were incubated at 28°C for 5–7 days and observed for the formation of a clear zone around the bacterial colonies (Louden et al., 2011).

### Hydrogen cyanide production

Bacteria were streaked into nutrient agar plates supplemented with glycine (Lorck, 1948). The lid was lined with filter paper (Whatman No. 1) impregnated with 1% picric acid and 10% of sodium carbonate. Petri plates were sealed with parafilm and incubated at 28 ± 1°C for 96 h in inverted condition. Discoloration of the filter paper from yellow to brown after incubation was considered as microbial production of hydrogen cyanide.

### Scoring of Plant Growth Promoting Bacteria (PGPB) isolates

After determining the PGP traits for all 36 isolates, a row/column matrix was generated (bacterial isolates in row and PGP traits in column) based on the score assigned to each isolate against each PGP trait. The score to each isolate against each trait was assigned as per the formula given below:


$$\text{Score}=\frac{\text{Sample value}-\text{Minimum value}}{\text{Maximum value}-\text{Minimum value}}$$

The score against each isolate for a particular trait was given in such a way that the highest trait value among 36 isolates got the maximum score 1 and the least trait got the score of 0. Finally, the scores of seven traits defined as cumulative or total score of multifaceted traits of an isolate was calculated.

### Statistical analysis

For each PGP parameter, the significant difference among means of isolates were determined by one-way ANOVA incorporating Levene’s test of homogeneity and test of normality. The multiple pairwise comparison among isolates’ means within a parameter was performed by Tukey’s honestly significant difference test (Tukey’s HSD) test at 95% confidence level (IBM SPSS v.21.0, SPSS Inc., Chicago, IL, USA).

The multivariate statistics was computed using PRIMER v 6.1.9 software (Primer-E Ltd, Plymouth, UK). The data matrix (10 different PGP traits as columns and bacterial isolates as rows) was normalized to eliminate the effects of different units, and square-root-transformed while performing principal component analysis (PCA). The Euclidean distance matrix was used as a measure of dissimilarity between isolates in terms of PGP traits. This data matrix was also subjected to hierarchical cluster analysis using group-average linking incorporating similarity of profile to test the significance difference at 95% confidence limit. Then, the hierarchical cluster profile was superimposed on the PCA plot to form ellipses (Thakuria et al., 2010).

### Molecular characterization of isolated microbes

The genomic Deoxyribonucleic acid (gDNA) of the isolated microbes were extracted as per procedure described by Thakuria et al. (2008). Post-extraction gDNA was analyzed on agarose gel electrophoresis, and the quality was checked using a Nano-drop^®^ 2000 spectrophotometer (Thermo Scientific, USA) based on A_260/230_ and A_260/280_ ratios. Extracted microbial DNA was amplified in a Gradient Master Cycler 5331 (Eppendorf Make, Germany) with a primer pair (27f and 1492r) specific to bacterial domain yields amplified product size of approximate 1,465 bp. The amplification reactions were carried out in a 25 µl volume containing 50 ng microbial DNA as a template, 100 nM of each oligonucleotide primers [27f (5’-AGAGTTTGATCCTGGCTCAG-3’) and 1492R (5’-GGTTACCTTGTTACGACTT-3’)], 12.5 µl of Go-Taq DNA polymerase (Promega, USA), and the required quantity of PCR grade water (HiMedia, India). The PCR reaction condition included an initial 3 min denaturation at 94°C, and was followed by 35 thermal cycles of 45 s at 94°C, 45 s at 56°C and 45 s at 72°C. Amplification was completed with the final extension step at 72°C for 7 min. All PCR products were examined by electrophoresed in an agarose gel (1% w v^-1^) using a 100 bp DNA ladder (New England Biolabs, UK). The amplified products were subjected to bi-directional sanger sequencing. Thus, obtained sequences were matched for homolog using the EzTaxon database and BLASTn database, NCBI. The accession numbers for sequences were obtained from the GenBank database. A phylogenetic consensus tree was constructed with reference sequences inferred from the Maximum Parsimony algorithm using MEGA v.11.

## Results

### Isolation and purification of bacterial microbiota from seeds

A total of 36 pure culture isolates were obtained in nutrient agar plates from surface-sterilized seeds of different citrus species collected from various regions of North East India. The different species with their respective microbial isolates obtained with their assigned name are listed in **Table 1**. The colonies obtained were selected on the basis of colony morphology and/or pigmentation and repeatedly streaked on fresh nutrient agar-media to obtain pure cultures. The purified cultures were maintained in nutrient agar slants, which were stored at 4°C, and glycerol stocks stored at -20°C. The morphological characteristics such as colony shape, size, color, elevation, margin, and surface were recorded at 48 h of growth on a nutrient agar plate and are presented in **Supplementary Table 1** and **Supplementary Figure 1**.

### Screening for multifaceted plant growth promotion properties

#### Production of indole acetic acid–like substance

**Figure 1:**
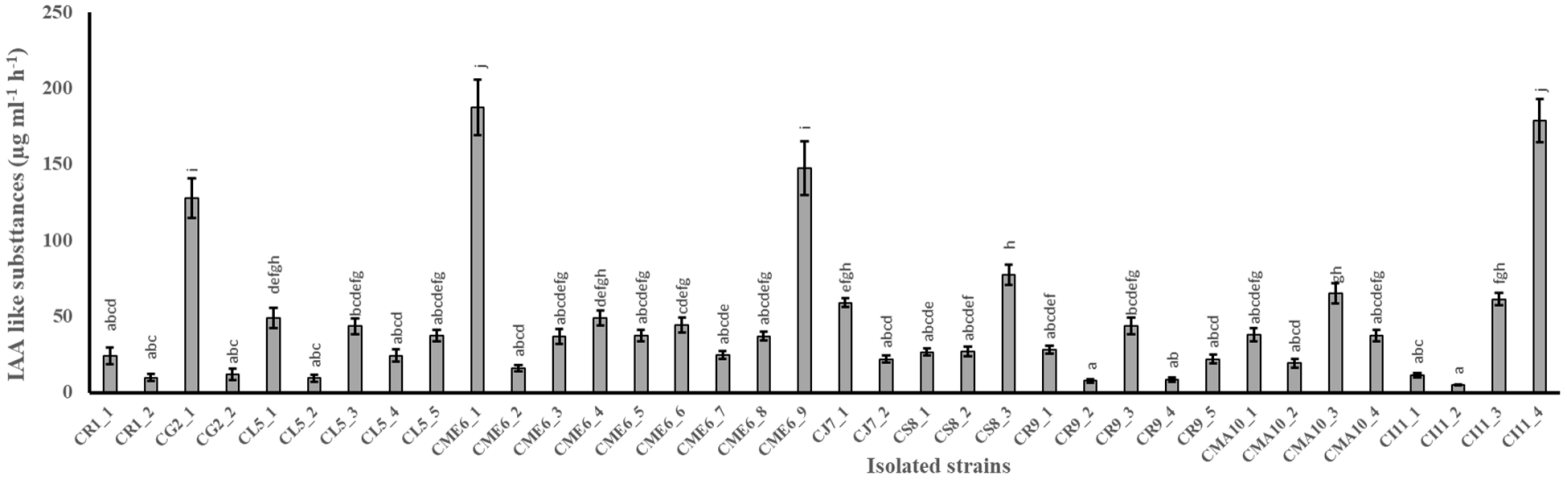
Production of indole acetic acid–like substances in tryptophan-amended nutrient broth media by citrus seed isolates. Within a column section, values that differ significantly (P < 0.05) are followed by different lower case letters, as determined by one-way analysis of variance (ANOVA) incorporating Tukey’s HSD test for pair-wise comparisons between means.

The quantitative production of IAA-like substances in tryptophan-amended nutrient broth media by 36 isolates is shown in **Figure 1**. The range of production of IAA-like substances varied from 4.8 ± 0.3 to 187.3 ± 18.1 µg ml^-1^ h^-1^ among the 36 isolates (**Table 2**). The maximum production of IAA-like substances was possessed by the isolate CME6-1 followed by CI11-4, CME6-9, and CG2-1 in the tune of 187.3, 178.8, 147.1, and 127.6 µg ml^-1^ h^-1^, respectively.

**Table 2:** Multifaceted plant growth–promoting (PGP) traits of citrus seed isolates.

Bacterial isolates	IAA production (μg ml^-1^ h^-1^)	Dissolution of insoluble phosphates (μg Pi ml^-1^ h^-1^)					Zinc complex solubilization efficiency (%)	
		Ca_3_(PO_4_)_2_	AlPO_4_	FePO_4_	Zn_3_(PO_4_)_2_	Na-Phytate	ZnO	ZnSO_4_
CR1_1	24.0 ± 5.3^abcd^	6.8 ± 0.2^d^	2.1 ± 0.2^ab^	1.8 ± 0.04^a^	2.4 ± 0.03^a^	12.7 ± 0.4^a^	–	–
CR1_2	9.6 ± 2.3^abc^	–	–	–	–	–	–	–
CG2_1	127.7 ± 12.8^i^	–	–	–	–	–	–	–
CG2_2	11.9 ± 3.7^abc^	–	–	–	–	–	–	–
CL5_1	49.2 ± 6.7^defgh^	1.1 ± 0.2^a^	–	–	6.0 ± 0.03^f^	–	–	–
CL5_2	9.3 ± 2.3^abc^	–	–	–	–	–	–	–
CL5_3	43.5 ± 5.2^bcdefg^	–	–	–	–	–	–	–
CL5_4	24.3 ± 3.9^abcd^	1.3 ± 0.2^a^	–	–	6.1 ± 0.04^f^	–	–	–
CL5_5	37.3 ± 3.6^abcdefg^	–	–	–	–	–	–	–
CME6_1	187.3 ± 18.1^j^	–	–	–	–	–	–	–
CME6_2	16.1 ± 1.9^abcd^	2.4 ± 0.3^ab^	–	–	–	–	–	–
CME6_3	37.0 ± 4.9^abcdefg^	3.4 ± 0.3^b^	1.7 ± 0.3a	–	4.9 ± 0.03^e^	12.2 ± 0.5^a^	87.6 ± 3.2^c^	150.4 ± 3.8^e^
CME6_4	49.1 ± 5.0^defgh^	14.8 ± 0.3^g^	3.6 ± 0.4b	2.8 ± 0.07c	13.2 ± 0.13^i^	18.0 ± 0.8^b^	40.2 ± 1.6^a^	175.9 ± 3.2^f^
CME6_5	37.6 ± 3.7^abcdefg^	9.7 ± 0.6^e^	2.5 ± 0.5^ab^	2.7 ± 0.04^c^	11.9 ± 0.10^h^	13.9 ± 0.9^a^	–	290.5 ± 5.7^g^
CME6_6	44.4 ± 5.2^cdefg^	–	–	–	–	–	–	–
CME6_7	24.9 ± 2.6^abcde^	–	–	–	–	–	–	–
CME6_8	37.0 ± 3.0^abcdefg^	11.8 ± 1.0^f^	2.3 ± 0.4^ab^	2.3 ± 0.04^b^	6.7 ± 0.05^g^	14.0 ± 0.6^a^	–	67.6 ± 2.1^a^
CME6_9	147.2 ± 17.7^i^	–	–	–	–	–	–	–
CJ7_1	59.0 ± 3.1^efgh^	3.8 ± 0.4^bc^	3.3 ± 0.5^ab^	2.3 ± 0.05^b^	5.8 ± 0.04^f^	12.7 ± 0.5^a^	111.7 ± 4.1^d^	–
CJ7_2	22.0 ± 2.4^abcd^	–	–	–	–	–	–	–
CS8_1	26.6 ± 2.5^abcde^	–	–	–	–	–	–	–
CS8_2	27.1 ± 3.2^abcdef^	–	–	–	–	–	–	–
CS8_3	77.4 ± 6.5^h^	2.6 ± 0.2^ab^	1.7 ± 0.3^a^	1.5 ± 0.02^a^	4.4 ± 0.03^d^	16.6 ± 0.5^b^	65.9 ± 2.5^b^	91.8 ± 2.3^b^
CR9_1	28.2 ± 2.6^abcdef^	–	–	–	–	–	–	–
CR9_2	7.6 ± 1.2^a^	4.1 ± 0.6^bc^	2.3 ± 0.4^ab^	2.9 ± 0.05^c^	2.8 ± 0.03^b^	–	90.3 ± 2.6^c^	61.4 ± 2.5^a^
CR9_3	43.8 ± 5.7^bcdefg^	–	–	–	–	–	–	–
CR9_4	8.5 ± 1.6^ab^	–	–	–	–	–	–	–
CR9_5	22.0 ± 3.0^abcd^	–	–	–	–	–	–	–
CMA10_1	37.9 ± 4.4^abcdefg^	2.5 ± 0.3^ab^	–	–	–	16.8 ± 0.4^b^	97.3 ± 5.1^c^	105.1 ± 2.5^c^
CMA10_2	19.2 ± 3.0^abcd^	–	–	–	–	–	–	–
CMA10_*3*	65.3 ± 6.7^gh^	5.5 ± 0.5^cd^	–	–	5.1 ± 0.06^e^	–		
CMA10_4	37.6 ± 3.8^abcdefg^	–	–	–	–	–	–	–
CI11_1	11.6 ± 1.5^abc^	–	–	–	–	–	–	–
CI11_2	4.8 ± 0.3^a^	–	–	–	–	–	–	–
CI11_3	61.3 ± 4.1^fgh^	6.2 ± 0.4^d^	2.9 ± 0.2^ab^	4.6 ± 0.2^d^	3.8 ± 0.09^c^	13.5 ± 0.4^a^	64.0 ± 2.0^b^	126.0 ± 3.0^d^
CI11_4	178.8 ± 14.0^j^	–	–	–	–	–	–	–

Within a column section, values that differ significantly (P < 0.05) are followed by different lower case letters, as determined by one-way analysis of variance (ANOVA) incorporating Tukey’s HSD test for pair-wise comparisons between means.

#### Nitrogen fixation

Out of 36 bacterial isolates, 13 bacterial isolates tested positive for nitrogen fixation by production of blue color from green in the nitrogen-free bromothymol blue medium (**Supplementary Figure 2**). The bacteria positive for N_2_ fixation included isolates CG2-1, CL5-1, CL5-5, CME6-1, CME6-5, CME6-8, CR9-1, CR9-3, CMA10-3, CMA10-4, CI11-2, CI11-3, and CI11-4.

#### Phosphate-solubilization ability test

The ability of all the seed microbial isolates to solubilize different insoluble phosphates and organic phosphate was examined in plate as well as liquid culture. The creation of a solubilization zone surrounding the colony demonstrated the isolates’ phosphate solubilization ability on Pikovskaya’s agar plates modified independently with different phosphate complexes (**Table 2**; **Supplementary Figure 3**). A total of 14 bacterial isolates were able to solubilize Pi (inorganic phosphate) on Ca_3_(PO_4_)_2_-amended Pikovskaya’s agar plates, and the range of dissolved inorganic phosphate in liquid culture was from to 1.31 ± 0.2 to 14.84 ± 0.3 µg Pi ml^-1^ h^-1^ with isolates CME6-4, CME6-8, and CME6-5 possessing the maximum ability for dissolution of tricalcium phosphate. There were 12 bacterial isolates that were able to solubilize Pi on the Zn_3_(PO_4_)_2_-amended Pikovskaya’s agar, and the range of dissolved inorganic phosphate was from 2.44 ± 0.03 to 13.16 ± 0.13 µg Pi ml^-1^ h^-1^ in which isolate CME6-4 possessed the maximum ability for dissolution of zinc phosphate followed by CME6-5 and CME6-8. In case of AlPO_4_-amended Pikovskaya’s agar plates, nine isolates were able to produce the solubilization zone around the colony to solubilize Pi with the range of dissolved inorganic phosphate was from 1.74 ± 0.3 to 3.61 ± 0.4 µg P_i_ ml^-1^ h^-1^ where isolate CME6-4 possessed the maximum ability for dissolution of aluminum phosphate followed by CJ7-1 and CI11-3. Nine bacterial isolates were able to produce a solubilization zone around the colony on FePO_4_-amended Pikovskaya’s agar, and the dissolved inorganic phosphate ranged from 1.54 ± 0.02 to 4.61 ± 0.2 µg Pi ml^-1^h^-1^ with isolate CI 11-3 possessing the maximum solubilization ability followed by CR 9-2 and CME 6-4. All bacterial isolates were also tested for the ability to release ortho-phosphate on sodium-phytate (Na-phytate)–amended Pikovskaya’s agar plates. Nine bacterial isolates were able to produce a solubilization zone around the colony (**Supplementary Figure 3E**). The range of ortho-phosphate released from the phytate complex was between 12.17 ± 0.5 and 18.00 ± 0.8 µg P_i_ ml^-1^ h^-1^ among nine isolates with the maximum ability to mineralize the phytate complex possessed by isolate CME6-4 followed by CMA10-1 and CS8-3.

**Figure 2:**
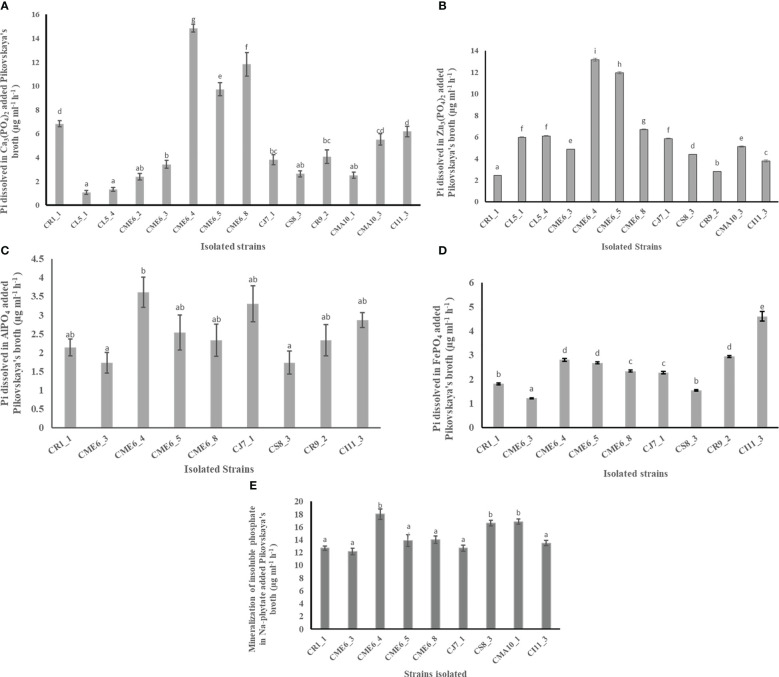
Phosphate solubilization ability of citrus seed isolates in Pikovskaya’s media amended with **(A)** Ca_3_(PO_4_)_2_), **(B)** Zn_3_(PO_4_)_2,_ **(C)** AlPO_4_, **(D)** FePO_4_, and **(E)** Na-phytate. Within a column section, values that differ significantly (P < 0.05) are followed by different lower case letters, as determined by one-way analysis of variance (ANOVA) incorporating Tukey’s HSD test for pair-wise comparisons between means.

The quantitative data on production of inorganic phosphate by the isolates having a solubilizing ability from Ca_3_(PO_4_)_2,_ Zn_3_(PO_4_)_2,_ AlPO4, FePO_4_, and Na-phytate amended separately in Pikovskaya’s broth is shown in **Figures 2A–E**, respectively.

### Dissolution of insoluble zinc complexes

**Figure 3:**
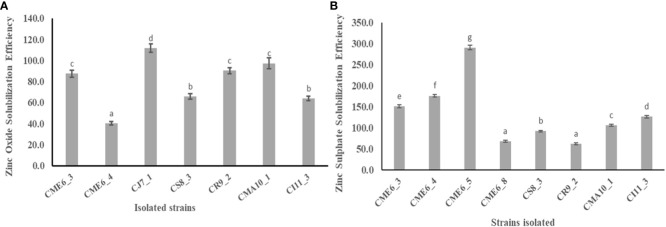
Zinc solubilization efficiency in Bunt and Rovira media amended with **(A)** 0.1% ZnO and **(B)** 0.1% ZnSO_4_. Within a column section, values that differ significantly (P < 0.05) are followed by different lower case letters, as determined by one-way analysis of variance (ANOVA) incorporating Tukey’s HSD test for pair-wise comparisons between means.

Solubilization zone development around the colony on zinc-amended Bunt and Rovira media concluded that the isolates have the potential to solubilize zinc. There were 7 of the 36 isolates that showed a positive result by the formation of a solubilization zone around the colony in the zinc oxide–amended Bunt and Rovira media (**Supplementary Figure 4A**). The zinc oxide– dissolving efficiency ranged from 40.2 ± 1.6 to 111.7 ± 4.1, with isolate CJ7-1 having the highest efficiency, followed by CMA10-1 and CR9-2. There were 8 of the 36 isolates that showed a positive result by the formation of a solubilization zone around the colony in the zinc sulfate–amended Bunt and Rovira media agar plates (**Supplementary Figure 4B**). The zinc sulfate dissolution efficiency ranged from 61.4 ± 2.5 to 290.5 ± 5.7, with isolate CME 6-5 having the highest efficiency followed by CME 6-4 and CME 6-3. The quantitative data on the zinc oxide solubilization efficiency and zinc sulfate solubilization efficiency is shown in **Figures 3A, B**, respectively (**Table 2**).

### Siderophore-like substance

**Figure 4:**
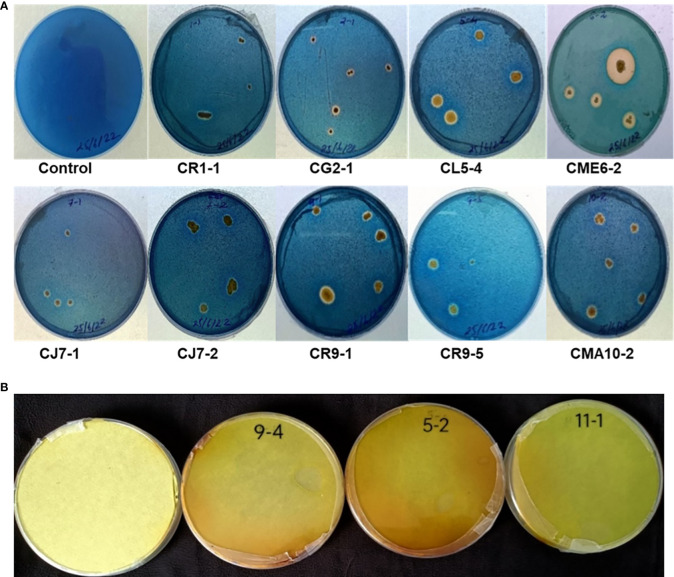
**(A)** Siderophore production. **(B)** HCN production.

The isolates were tested for siderophore production on CAS blue agar medium. There were 9 bacterial isolates, *viz.* CR1-1, CG2-1, CL5-4, CME6-2, CJ7-1, CJ7-2, CR9-1, CR9-5, and CMA10-1, out of the 36 bacterial isolates that showed a positive result for siderophore production on CAS agar medium by producing a halo zone around the colony as presented in **Figure 4A**.

### HCN production

Only three isolates, *viz.* CL5-2, CR9-4, and CI11-1, out of the 36 bacterial isolates that showed positive results for HCN production as the filter paper turned reddish brown from yellow as depicted in **Figure 4B**.

### Scoring of PGPB isolates and statistical analysis

**Figure 5:**
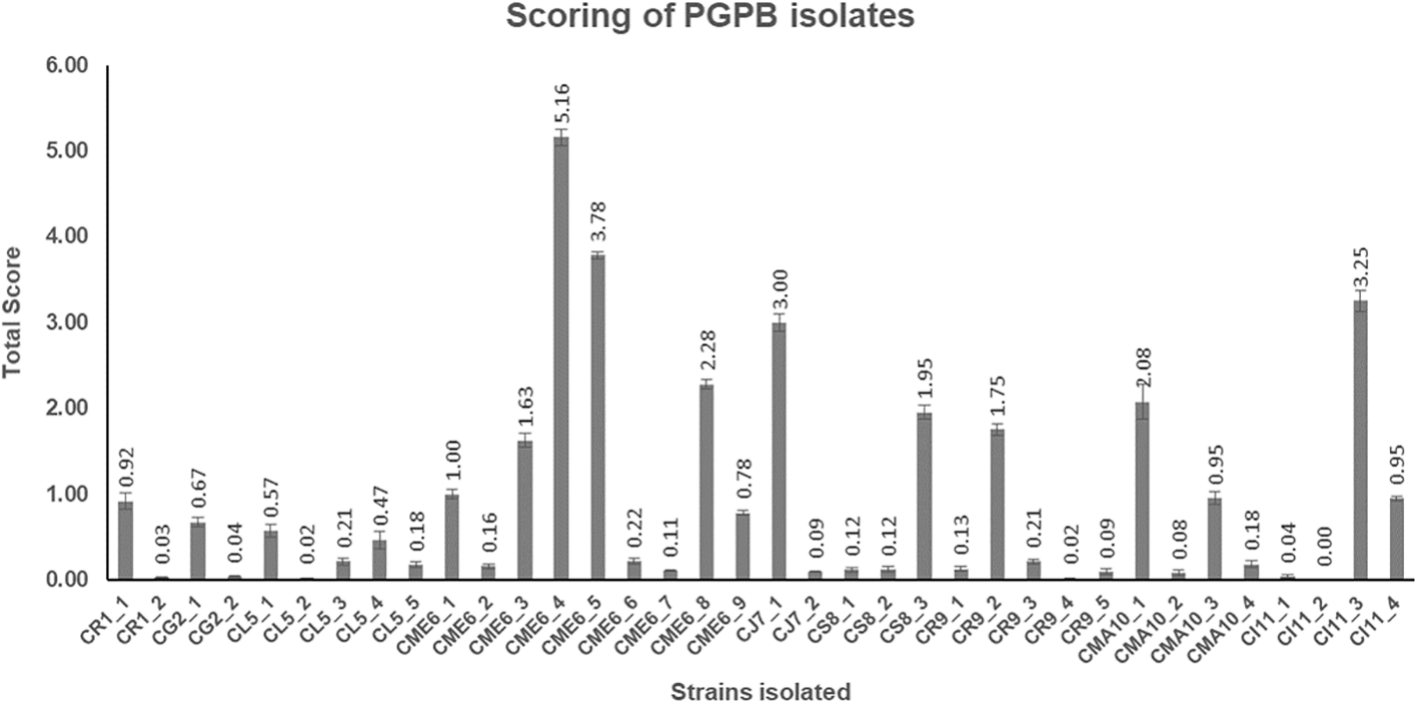
Total score obtained by each microbial isolate on the basis of their multifaceted plant growth–promoting (PGP) properties.

All the 36 microbial isolates were scored for the different PGP properties including production of IAA-like substances, dissolution of insoluble phosphates [Ca_3_(PO_4_)_2_, AlPO_4_, FePO_4_, Zn_3_(PO_2_)_4_, and Na-phytate], and dissolution of insoluble zinc complexes (ZnO and ZnSO_4_). The data on the total (cumulative) score of each of the 36 isolates are presented in **Figure 5** (**Supplementary Table 2**). The isolate CME6-4 (5.16) got the highest score followed by CME6-5 (3.78), CI11-3 (3.25), CJ7-1 (3.00), CME6-8 (2.28), and CMA10-1 (2.08).

**Figure 6:**
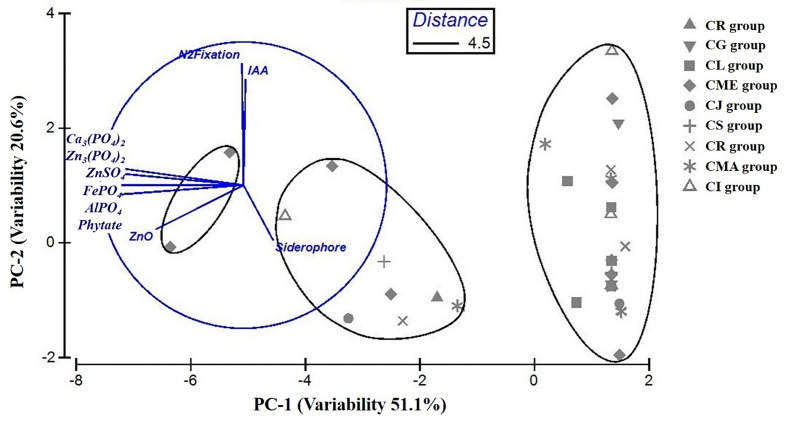
Comparison of variability in PGP traits among bacterial isolates obtained from nine different citrus species performed by principal component analysis (PCA). Ellipses represent superimposed hierarchical clusters (Euclidean distance 4.5) based on the Euclidean distance matrix, deduced using group-average linking incorporating similarity of profile at 95% confidence limit.

**Figure 7:**
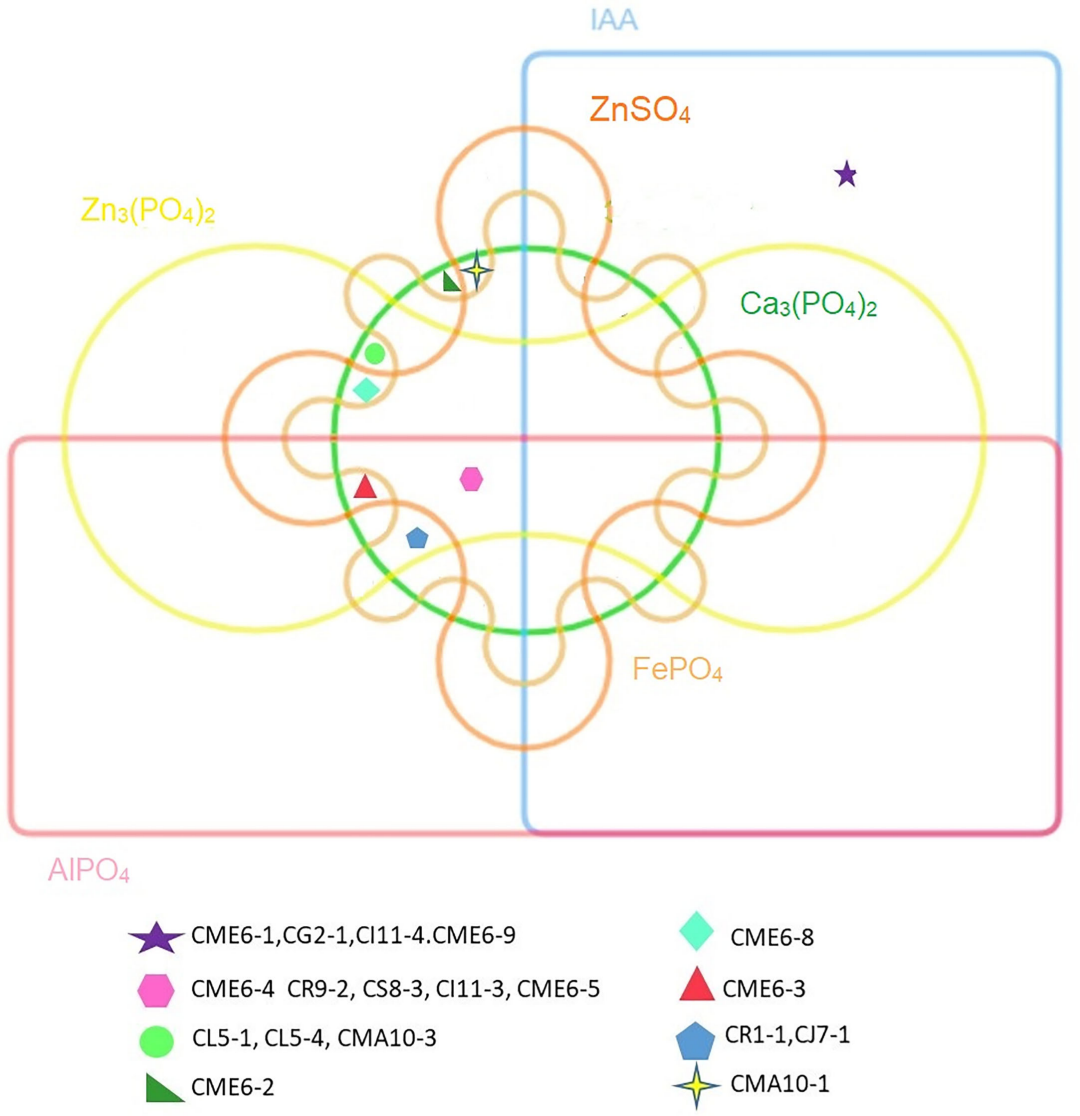
Venn diagram showing microbial isolates having common PGP properties.

The statistical analysis performed using PCA showed the multivariate interaction between the PGP traits. There were three distinct clusters separated along PC axis 1 that explained 51.1% variability in terms of PGP traits (**Figure 6**). The variables, namely, dissolution of Ca_3_(PO_4_)_2_, Zn_3_(PO_4_)_2_, ZnSO_4_, FePO_4,_ and AlPO_4_ and mineralization of Na-phytate contributed significantly in formation of clusters along PC axis 1. On the other hand, production of IAA-like substances and N_2_-fixation contributed significant variability (20.6%) in separating bacterial isolates along PC axis 2. The coefficients in the linear combinations of variables making up PC axis 1 were dissolution of Ca_3_(PO_4_)_2_, Zn_3_(PO_4_)_2_, ZnSO_4_, FePO_4,_ AlPO_4_, and mineralization of Na-phytate and PC axis 2 were production of IAA-like substances and N_2_-fixation (**Table 3**). The Venn diagram in **Figure 7** shows the microbial isolates from citrus seeds that exhibit common PGP features such as phosphate solubilization, zinc sulfate solubilization, and IAA production.

**Table 3:** Coefficients in the linear combinations of variables (PGP traits) making up principal component axes in the principal component plot.

PGP traits of bacterial isolates	PC-1	PC-2
Dissolution of Ca_3_(PO_4_)_2_	-0.396	0.067
Dissolution of Zn_3_(PO_4_)_2_	-0.376	0.093
Dissolution of ZnSO_4_	-0.372	0.063
Dissolution of ZnO	-0.272	-0.248
Dissolution of FePO_4_	-0.386	0.000
Dissolution of AlPO_4_	-0.419	-0.058
Mineralization of Na-phytate	-0.396	-0.053
Siderophore activity	0.092	-0.310
N_2_-fixation activity	-0.005	0.682
IAA-like substance production	0.007	0.595

### Molecular characterization of isolated strains

**Figure 8:**
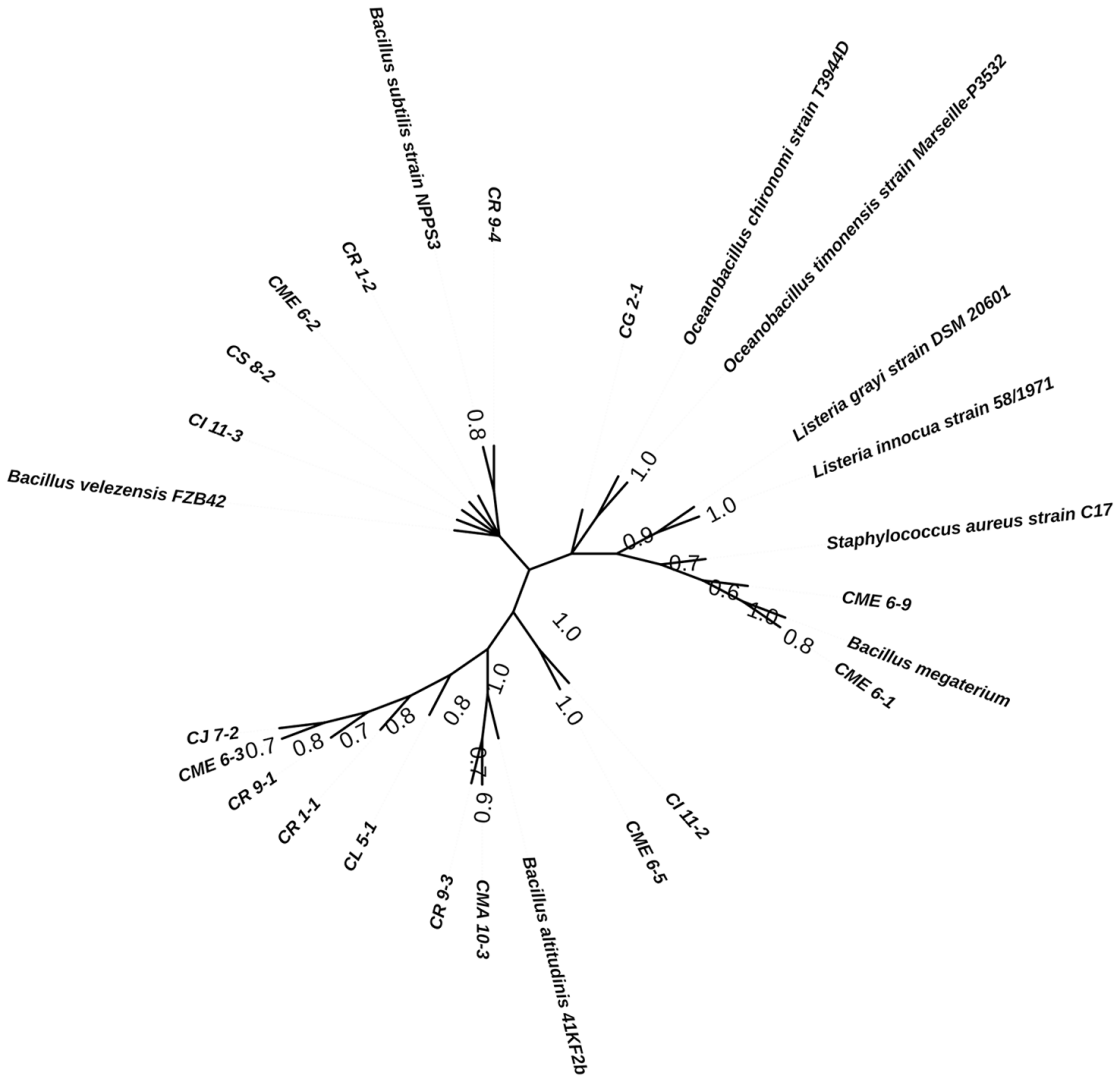
Phylogenetic consensus tree for the isolated strains with reference sequences inferred from the Maximum Parsimony algorithm using MEGA v.11.

Bacterial isolates characterized for multiple PGP traits were selected for sequencing of 16S rRNA genes for determination of identity. The bacterial isolates were found to have homology with the genus *Bacillus* and are listed in **Table 4**. The sequence information was submitted to the GenBank accession. The consensus phylogenetic tree constructed with the nearest member of the genus *Bacillus* is depicted in **Figure 8** along with the bootstrap value.

**Table 4:** Phylogeny of selected culturable bacterial isolates obtained from the seeds of citrus species of the northeastern region.

Strain	%homology with respect to 16S rRNA gene homology	Putative identity of bacterium (if known)	GenBank accession no.
CR1-1	98.23	*Bacillus altitudinis*	OP693617
CR1-2	97.94	*Bacillus amyloliquefaciens*	OP693619
CG2-1	99.00	*Kocuria rhizophila*	OP696594
CL5-1	99.00	*Bacillus altitudinis*	OP684790
CME6-1	96.04	*Priestia megaterium*	OP696618
CME6-2	98.78	*Bacillus siamensis*	OP693637
CME6-3	99.12	*Bacillus altitudinis*	OP696652
CME6-5	98.84	*Bacillus altitudinis*	OP686474
CME6-9	95.10	*Priestia flexa*	OP686476
CJ7-2	98.13	*Bacillus subtilis*	OP694174
CS8-2	96.93	*Bacillus velezensis*	OP686477
CR9-1	99.24	*Bacillus* sp.	OP695777
CR9-3	99.02	*Bacillus altitudinis*	OP692732
CR9-4	99.77	*Bacillus subtilis*	OP696578
CMA10-3	99.52	*Bacillus altitudinis*	OP696654
CI11-2	99.93	*Bacillus altitudinis*	OP692736
CI11-3	99.50	*Bacillus velezensis*	OP692753

## Discussion

The Food and Agriculture Organization of the United Nations estimated that during the past century, agricultural crops have lost approximately 75% of their genetic diversity because of widespread desertion of genetically diverse traditional crops in favor of genetically uniform modern crop types. According to the concept of a “plant holobiont,” a plant is a coevolved species conglomerate made up of an interkingdom community of bacterial, archaeal, fungal, and other eukaryotic species (Vandenkoornhuyse et al., 2015). The domestication and cultivation of agricultural plants may have a detrimental effect on the beneficial microbial community of plants. According to Pérez-Jaramillo et al. (2016), the “back to the roots” outlook calls for investigating the microbiomes of wild relatives of agricultural plant species and their natural environments in order to identify beneficial bacteria that may have been lost during domestication and reintroduce them in seeds of current crop species. The northeastern region is renowned as a treasure house of citrus germplasm and is home to several landraces of the citrus species, many of which remain unexploited due to the lack of commercial cultivation (Singh and Singh, 2006). In the current work, healthy fruits of various citrus species were collected from different parts of northeastern India. Microbial strains were isolated from the surface-sterilized seeds of the fruits, which were further screened for features that promote plant growth. 16S rRNA–based molecular characterization was carried out for taxonomic identification of the seed microbiome.

A total of 36 microbial isolates were recovered from seeds of several citrus species, including 2 from Sikkim orange seeds, 2 from pummelo seeds, 5 from khasi papeda seeds, 9 from sour pummelo seeds, 2 from rough lemon seeds, 3 from sweet orange seeds, 5 from khasi mandarin seeds, 4 from melamesian papeda seeds, and 4 from Indian wild orange seeds. The colony isolates displayed a variety of shapes, of which the majority were circular, while a small number were irregular, most had glistening appearances and dull appearances were rare, flat to elevated surfaces, and white colony colors for the majority of strains, with cream, yellow, and orange pigments for a few. Faddetta et al. (2021) reported 60 endophytes from *C. limon* seeds that were streaked on a nutrient agar plate on the basis of distinctive colony morphology and pigmentation. Numerous studies have reported the association of microorganisms with the seeds of various crops like maize, rice, wheat, rice cut grass, beans, chickpea, cucurbits, and tomato (Johnston-Monje and Raizada, 2011; Verma et al., 2017;. Herrera et al., 2016; Khalaf and Raizada, 2016; Walitang et al., 2017; Morella et al., 2019). Different strains of epiphytic bacteria such as *Bacillus amyloliquefaciens* and *Pseudomonas parafulva* have been found to be associated with the fruits of several citrus species, including *C. unshiu, C. reticulata*, and *C. sinensis* and tend to have positive effect on plant health (Jing et al., 2023). PGPR isolated from the rhizospheres of different citrus species was prepared in an aqueous suspension and then used as a foliar spray and soil-drench, which led to increased crop growth (Chakraborty et al., 2016).

The auxin family member that has been investigated the most is indole-3-acetic acid, and tryptophan is the primary IAA precursor in five of the major pathways used by bacteria to produce auxin (Gamalero and Glick, 2011). The primary effects of bacterial IAA on plants include increased lateral and adventitious rooting, which improves nutrient uptake and root exudation, which, in turn, promotes bacterial growth on the roots (Thakuria et al., 2004). The synthesis of IAA-like compounds by the 36 isolates in the current investigation ranged from 4.8 ± 0.3 to 187.3 ± 18.1 µg ml^-1^ h^-1^ in the presence of L-tryptophan as a precursor among which CME6-1, CI11-4, CME6-9, and CG2-1 are the highest producers. The results were consistent with those of Verma et al. (2017), who reported that two isolates of rex rice (*Oryza sativa* L.) seed endophytic bacteria, *E. asburiae* and *P. dispersa*, produced 70.81 ± 0.98 and 7.98 ± 0.58 µg ml^-1^ IAA, respectively. Faddetta et al. (2021) reported IAA production ability by three microbial endophytes isolated from *C. limon* seeds. Several other researchers have also noted the capacity of seed microbial endophytes to produce IAA in a variety of crops, including cucurbits (Khalaf and Raizada, 2016), rice (Walitang et al., 2017), marama beans (Chimwamurombe et al., 2016), maize (Sandhya et al., 2017), and wheat (Herrera et al., 2016).

Phosphate solubilization is one of the most crucial bacterial physiological features in soil biogeochemical cycles and in plant growth promotion by PGPB. In the current study, the capacity of dissolution of insoluble phosphates was determined on Pikovskaya’s media amended individually with various inorganic phosphate complexes, which include Ca_3_(PO_4_)_2,_ AlPO_4,_ FePO_4_ and Zn_3_(PO_4_)_2._ In the Ca_3_(PO_4_)_2_-amended Pikovskaya’s agar plate, 14 isolates produced solubilization zones with solubilization ranging from 1.31 ± 0.2 to 14.84 ± 0.3 µg P_i_ ml^-1^ h^-1^. Nine bacterial isolates created a solubilization zone with a degree of solubilization between 1.54 ± 0.02 and 4.61 ± 0.2 µg P_i_ ml^-1^ h^-1^ in FePO_4_-amended Pikovskaya’s media, while nine bacterial isolates produced a solubilization zone with a degree of solubilization ranging from 1.74 ± 0.3 to 3.61 ± 0.4 µg P_i_ ml^-1^ h^-1^ in AlPO_4_-amended Pikovskaya’s media. The Zn_3_(PO_4_)_2_ dissolution ranged between 2.44 ± 0.03 and 13.16 ± 0.13 µg P_i_ ml^-1^ h^-1^ among 12 isolates that showed a solubilization zone in Zn_3_(PO_4_)_2_-amended Pikovskaya’s agar plate. The findings were supported by Thokchom et al. (2014), who reported that on Ca_3_(PO_4_)_2_-supplemented medium, 54.8% of mandarin orange rhizobacterial strains could solubilize insoluble P with phosphate solubilization ranging from between 6.2 ± 1.1 and 267.9 ± 24.1 µg ml^-1^. The ability of seed endophytes to solubilize phosphate has been documented in numerous papers Endophytes isolated from Rex Rice seed was found with phosphate solubilization properties (Verma et al., 2017). Johnston-Monje and Raizada (2011) reported 63 isolates obtained from *Zea mays* that solubilized phosphate. Seed-associated endophytes of cucurbit family reported 21% of strains, primarily *Bacillus*, lactic acid bacteria and *Enterobacteriaceae*, showed phosphate solubilization on Ca_3_(PO_4_)_2_-amended plates (Khalaf and Raizada, 2016). The outcomes also agreed with those of Faddetta et al. (2021), who discovered that bacteria cultivated from *C. limon* seeds could flourish on solid media containing various sources of inorganic phosphate (Ca-, Fe-, and Al-). *Gluconacetobacter diazotrophicus* was reported to solubilize Zn_3_(PO_4_)_2_ (Saravanan et al., 2007). The primary strategy used by phosphate- solubilizing bacteria to dissolve inorganic P is the production of low-molecular-weight organic acids, which bind phosphate with their hydroxyl and carboxyl groups, chelating cations, and contributing soil acidification, both of which result in the release of soluble phosphate (Rodriguez et al., 2004). Phosphorus is stored in plant seeds and pollen in form of phytate as a primary inositol source. Many seeds, notably those from grains and legumes, store phosphorus mostly in the form of phytate, which promotes the growth of seed endophytes during germination. Bean seed phytate solubilizers include *Acinetobacter, Bacillus, Streptomyces*, and *Rhizobium* endophyticum (López-López et al., 2010). Three bacteria isolated from surface-sterilized seeds of rice have been reported to act as phytate solubilizers (Verma et al., 2017), and isolated seed microbial endophytes of *C. limon* were able to grow on organic phosphate, i.e., Na-phytate-amended agar plates (Faddetta et al., 2021). The results of the current study revealed nine isolates with solubilizing capacities for sodium phytate ranging from 12.17 ± 0.5 to 18.00 ± 0.8 µg P_i_ ml^-1^ h^-1^.

Citrus decline is thought to be a condition with numerous contributing variables. However, a clear causal involvement of zinc nutrition as a contributor to citrus decline/blight have been hypothesized by many. Zinc-solubilizing bacteria are potential substitutes for zinc supplements because they transform applied inorganic zinc into usable forms. In this study, the zinc solubilization efficiency of bacterial isolates was investigated on modified Bunt and Rovira medium amended separately with two insoluble zinc sources, namely, ZnO and ZnSO_4._ Seven isolates were able to solubilize ZnO (solubilization efficiencies ranged from 64 to 111.68), with isolate CJ7-1 having the largest solubilization diameter of 86 mm. Eight isolates solubilize ZnSO4 (solubilization efficiencies ranged from 60.4 to 290.45, with the isolate CME6-5 (*B. altutidinis*) having the largest solubilization diameter of 164.4 mm. The findings were consistent with those of Saravanan et al. (2004) who reported 17 bacterial isolates with strain ZSB-O-1 (*Bacillus* sp.) producing a clearing zone of 2.80 cm on ZnSO_4_ and 1.80 cm of clearing zone with ZnO-containing medium, and *G. diazotrophicus* strain produced a solubilization zone diameter of 36 mm (Saravanan et al., 2007).

Since nitrogen makes up approximately 1.5%–2.0% of a plant’s dry matter and 16% of its total protein, plants need more nitrogen than other organisms. Different parts of plant act as microniches for the colonization of endophytic diazotrophs, where without competition with other microorganisms and absence of soil limitations present in the rhizosphere, the endophytes fix nitrogen (Verma et al., 2017). In the present study, 36% of the total microbe isolates were able to fix N_2_ in N-free bromothymol blue media distinguished by a shift in the medium’s color from green to blue. Other studies have also reported the N_2_ fixation ability by seed endophytes of *Tylosema esculentum* (Chimwamurombe et al., 2016), cucurbit family (Khalaf and Raizada, 2016) and *Zea* species (Johnston-Monje and Raizada, 2011).

Siderophore production is a vital characteristic of endophytic bacteria. These bacteria enhance plant growth and health as they synthesize siderophores that chelate iron in an iron-limited condition making it accessible for the plants and unavailable for phytopathogens (Mercado-Blanco and Bakker, 2007). In the present study, nine isolates of the total isolated strains were able to synthesize siderophores on CAS blue agar medium evident by formation of a halo zone around the colony. The findings were in line with the results of Chimwamurombe et al. (2016) who reported 13 isolates of *T. esculentum* seed positive for siderophore production and Herrera et al. (2016) who found six bacterial strains isolated from wheat seed positive for siderophore production.

HCN toxicity is influenced by its capacity to inhibit cytochrome c oxidase as well as other significant metalloenzymes (Nandi et al., 2017). While the ability of bacteria to make HCN is often considered as a negative factor, it has been suggested that HCN-producing endophytic bacteria may have important applications for the biocontrol of weeds, soil phytopathogenic fungi, and nematodes. In this study, only three isolates produced HCN in amounts that were positive. *Phragmites australis* seed endophytes (Verma et al., 2018), maize seed and root endophytes (Sandhya et al., 2017), and endophytes from nodules and roots of chickpea and pea (Maheshwari et al., 2019) have all been documented to be capable of producing HCN.

A fascinating feature in the current study is that the genus *Bacillus* appears to make up the culturable core seed microbiota across all the examined citrus species. A distinctive characteristic of seed endophytes seen in prior studies is the capacity to produce endospores, which are assumed to protect the inhabitants of the seed from internal changes (Truyens et al., 2015). Munir et al. (2020) reported that *B. velezensis, Curtobacterium luteum, Microbacterium testaceum*, and *B. subtilis* as the most prevalent endophytes in the leaves of 24 citrus types, with *B. subtilis* occurring most frequently in healthy plants. In the leaf tissues of numerous citrus rootstocks, the predominated bacterial species was found to be *Pantoea agglomerans* and *B. pumilus* (Araújo et al., 2001). *Proteobacteria, Actinobacteria, Bacteroidetes*, and *Firmicutes* were the most prevalent bacterial phyla in the microbial endophytic community of *C. limon* seeds. (Faddetta et al., 2021). Further, the genus *Bacillus* has been reported as common member of seed microbiota in different crops such as rice (Cottyn et al., 2001), gurana (e Silva et al., 2016), beans (López-López et al., 2010), and cucurbit family (Khalaf and Raizada, 2016). Members of the genus *Bacillus* is functionally diverse and one of the most widely utilized bacteria in the field of agrobiotechnology. It is well known that members of the genus *Bacillus* possess a multitude of beneficial attributes that aid plants either directly or indirectly by acquisition of nutrients, phytohormone production, and shielding plants from biotic and abiotic stress. In this study, various isolates obtained from the citrus seed belonging mainly to the *Bacillus* genus showed multifaceted plant growth promotion traits like nitrogen fixation, phosphate and zinc solubilization, and IAA production. Nitrogen fixation ability has been reported by *B. pumilus* and *B. altitudinis* that were isolated from the rhizosphere of maize and rice, respectively (Habibi et al., 2014). There were 16 of the 19 distinct *Bacillus* strains screened for the nifH gene that were found to be positive (Yousuf et al., 2017). According to Sharma et al. (2013), many species of *Bacillus* including *B. megaterium* M510, which was isolated from the maize rhizosphere in the eastern Himalayan region, were found to be capable of solubilizing iron and aluminum phosphate in addition to moderately solubilizing tricalcium phosphate. *B. subtilis, B. thuringiensis, B. aryabhattai*, and *B. tequilensis* are well-known zinc solubilizers (Singh et al., 2017). The isolates isolated from the chickpea rhizosphere that were highly similar to *B. altitudinis* displayed PGP traits including IAA, ammonia, siderophores, and phosphate solubilization (Kushwaha et al., 2021)*. K. rhizophila*, isolated from citrus seed in this study was found to be a high IAA producer, while *K. rhizophila Y1* isolated from maize rhizosphere soil exhibited growth-promoting characteristics such as phosphate solubilization and IAA synthesis (Li et al., 2020).

Microbial strains with PGP properties have the potential to be used in the development of bioinoculants, biofertilizers, and biopreparations that improve crop plant growth and yield. They are committed to being used in organic/natural farming as they increase crop yields enhancing the uptake of nutrition and improving plant vigor, combat pathogens, and increase plant abiotic resistance while conferring habitat-adapted tolerance to the host plant (Pylak et al., 2019). Endophytic isolates were evaluated as bioinoculants for potato tubers and recommended as biofertilizers to reduce dependency on chemical fertilizers (Kuźniar et al., 2019). Works have been focused on the evaluation of compatibility of the different PGP strains and prospecting the optimal combinations of the isolates to develop into bioinoculants for commercialization. *Bacillus* strains have been most frequently utilized as the common commercially available bioinoculants till date (Kuźniar et al., 2019). For instance, *Bacillus* species have successfully been used as priming agents in crops such as potato (Gururani et al., 2013), rice, mung bean, and chickpea (Chakraborty et al., 2011). As such, additional research is needed to translate the *in vitro* PGP activities of the isolates into pot experiments in greenhouse and field trials for improved crop yield.

## Conclusion

Our study clearly demonstrated that citrus seeds are an important source of beneficial seed microbiomes and that members belonging to genus *Bacilli* seem to be the core member as common dominant bacteria across eight citrus species tested. The culturable members of the citrus seed microbiome possessed multifaceted PGP traits like dissolution of insoluble phosphate and zinc complexes, mineralization of the organic phosphate complex, production of IAA-like substances, siderophore-like substances and HCN, and nitrogen fixation. Isolates CG2-1, CME6-1, CME6-4, CME6-5, CME6-8, CME6-9, CJ7-1, CMA10-1, CI11-3, and CI11-4 are promising candidate bioinoculants for development of microbial consortium having multifaceted PGP traits for nutritional benefits of nitrogen, phosphorus, and zinc and IAA hormonal benefits to citrus crops for better fitness in acid soils. Bacterial seed endophytes may be highly conserved in plant species to specific regions, necessitating the exploration of species from different regions. Additionally, research can be conducted using different growth media other than LB to increase the number of cultivable seed microbiomes from different citrus species. The isolated beneficial strains and their consortium will be tested further in greenhouse and field trials under natural environmental conditions.

## Data availability statement

The original contributions presented in the study are included in the article/**Supplementary Material**, further inquiries can be directed to the corresponding author.

## Author contributions

DT conceived and designed the study, validated results, and supervised the final draft of manuscript. SS, CC, and PU executed the experiments and wrote the first draft of manuscript. SH, PD, AS, and BL helped in citrus sample collection and checking the draft manuscript. All authors contributed to the article and approved the submitted version.
