# Supplementary material for: Plant growth–promoting traits of culturable seed microbiome of citrus species from Purvanchal Himalaya
Source: Front Plant Sci. 2023 Jul 10;14:1104927. doi: 10.3389/fpls.2023.1104927 (PMC10365123; doi:10.3389/fpls.2023.1104927)
Supplement: Supplementary file 1 [file DataSheet_1.docx]

**Plant growth promoting traits of culturable seed microbiome of citrus species from Purvanchal Himalaya**

Sakshi Sinha^a^, Dwipendra Thakuria^a,^*, Chayanika Chaliha^a^, Panchali Uzir^a^, Samarendra Hazarika^b^, Pranab Dutta^c^, A. K. Singh^a^, Bingiala Laloo^d^

^a^ School of Natural Resource Management, College of Post Graduate Studies in Agricultural Sciences, Central Agricultural University (Imphal), Umiam, Meghalaya, 793103, India.

^b^ Division of System Research and Engineering, ICAR Research Complex for North Eastern Hill Region, Umiam, 793103, Meghalaya, India.

^c^ School of Crop Protection, College of Post Graduate Studies in Agricultural Sciences, Central Agricultural University (Imphal), Umiam, Meghalaya, 793103, India.

^d^ School of Crop Improvement, College of Post Graduate Studies in Agricultural Sciences, Central Agricultural University (Imphal), Umiam, Meghalaya, 793103, India.

*****Correspondence

Prof. Dwipendra Thakuria

[thakuria.dwipendra@yahoo.co.in](mailto:thakuria.dwipendra@yahoo.co.in)

**SUPPLEMENTARY TABLE 1. The colony morphology of microbial isolates obtained from seeds of different citrus species**

| **Citrus species** | **Isolates** | **Colony morphology on NA plate** | | | | | |
| --- | --- | --- | --- | --- | --- | --- | --- |
|  |  | **Shape** | **Size**  **(mm)** | **Colour** | **Elevation** | **Margin** | **Surface** |
| ***C. reticulata*** | CR1-1 | Circular | 2 | White | Elevated | Entire | Smooth |
|  | CR1-2 | Irregular | 4 | White | Flat | Undulate | Smooth |
| ***C. grandis*** | CG2-1 | Circular | Pinpoint | Yellow | Elevated | Entire | Smooth |
|  | CG2-2 | Irregular | 3 | White | Flat | Undulate | Smooth |
| ***C.latipes*** | CL5-1 | Circular | 3 | White | Flat | Entire | Smooth |
|  | CL5-2 | Irregular | 5 | White | Flat | Undulate | Smooth |
|  | CL5-3 | Circular | 2 | White | Elevated | Entire | Smooth |
|  | CL5-4 | Circular | 1 | White | Flat | Entire | Smooth |
|  | CL5-5 | Circular | 2 | white | Elevated | Entire | Smooth |
| ***C.megaloxycarpa*** | CME6-1 | Circular | 4 | White | Elevated | Entire | Smooth |
|  | CME6-2 | Irregular | 5 | White | Flat | Filamentous | Smooth |
|  | CME6-3 | Circular | 2 | White | Elevated | Entire | Smooth |
|  | CME6-4 | Circular | 4 | White | Elevated | Entire | Smooth |
|  | CME6-5 | Circular | Pinpoint | White | Flat | Entire | Smooth |
|  | CME6-6 | Circular | 2 | Orange pigment | Elevated | Entire | Smooth |
|  | CME6-7 | Circular | Pinpoint | White | Flat | Entire | Smooth |
|  | CME6-8 | Circular | Pinpoint | White | Elevated | Entire | Smooth |
|  | CME6-9 | Irregular | 4 | Buff pigment | Elevated | Undulate | Wrinkled |
| ***C.jambhiri*** | CJ7-1 | Circular | 1 | White | Elevated | Undulated | Smooth |
|  | CJ7-2 | Circular | 3 | White | Flat | Entire | Smooth |
| ***C.sinensis*** | CS8-1 | Circular | 4 | White | Elevated | Entire | Smooth |
|  | CS8-2 | Irregular | 4 | White | Flat | Undulate | smooth |
|  | CS8-3 | Circular | 3 | White | Elevated | Entire | Smooth |
| ***C.reticulata*** | CR9-1 | Circular | Pinpoint | White | Elevated | Entire | Smooth |
|  | CR9-2 | Circular | 4 | White | Elevated | Entire | Smooth |
|  | CR9-3 | Circular | Pinpoint | White | Elevated | Entire | Smooth |
|  | CR9-4 | Irregular | 4 | White | Flat | Undulate | Dull |
|  | CR9-5 | Circular | 3 | Cream | Elevated | Undulate | Smooth |
| ***C. macroptera*** | CMA10-1 | Circular | 1 | White | Elevated | Entire | Smooth |
|  | CMA10-2 | Circular | 4 | White | Elevated | Undulate | Smooth |
|  | CMA10-3 | Circular | 2 | White | Elevated | Entire | Smooth |
|  | CMA10-4 | Circular | 2 | White | Elevated | Undulate | Smooth |
| ***C.indica*** | CI11-1 | Circular | 4 | White | Flat | Undulate | Smooth |
|  | CI11-2 | Circular | 1 | White | Elevated | Entire | Smooth |
|  | CI11-3 | Circular | 3 | White | Elevated | Entire | Smooth |
|  | CI11-4 | Circular | 2 | White | Elevated | Entire | Smooth |

**SUPPLEMENTARY TABLE 2. Score of microbial isolates of citrus seed based on their multifaceted PGP traits**

| **Microbe isolates** | **IAA-like substances (µg ml^-1^ h^-1^)** | **Dissolution of insoluble phosphate (µg ml^-1^ h^-1^ )** | | | | | **Dissolution of insoluble Zn complex** | | **Total score** |
| --- | --- | --- | --- | --- | --- | --- | --- | --- | --- |
|  |  | **Ca_3_(PO_4_)_2_** | **AlPO_4_** | **FePO_4_** | **Zn_3_(PO_4_)_2_** | **Na- Phytate** | **ZnO** | **ZnSO_4_** |  |
| CR1_1 | 0.11 | 0.42 | 0.22 | 0.08 | 0.00 | 0.09 |  |  | 0.92 |
| CR1_2 | 0.03 |  |  |  |  |  |  |  | 0.03 |
| CG2_1 | 0.67 |  |  |  |  |  |  |  | 0.67 |
| CG2_2 | 0.04 |  |  |  |  |  |  |  | 0.04 |
| CL5_1 | 0.24 | 0.00 |  |  | 0.33 |  |  |  | 0.57 |
| CL5_2 | 0.02 |  |  |  |  |  |  |  | 0.02 |
| CL5_3 | 0.21 |  |  |  |  |  |  |  | 0.21 |
| CL5_4 | 0.11 | 0.02 |  |  | 0.34 |  |  |  | 0.47 |
| CL5_5 | 0.18 |  |  |  |  |  |  |  | 0.18 |
| CME6_1 | 1.00 |  |  |  |  |  |  |  | 1.00 |
| CME6_2 | 0.06 | 0.10 |  |  |  |  |  |  | 0.16 |
| CME6_3 | 0.18 | 0.17 | 0.00 |  | 0.23 | 0.00 | 0.66 | 0.39 | 1.63 |
| CME6_4 | 0.24 | 1.00 | 1.00 | 0.41 | 1.00 | 1.00 | 0.00 | 0.50 | 5.16 |
| CME6_5 | 0.18 | 0.63 | 0.43 | 0.37 | 0.89 | 0.29 |  | 1.00 | 3.78 |
| CME6_6 | 0.22 |  |  |  |  |  |  |  | 0.22 |
| CME6_7 | 0.11 |  |  |  |  |  |  |  | 0.11 |
| CME6_8 | 0.18 | 0.78 | 0.33 | 0.26 | 0.40 | 0.31 |  | 0.03 | 2.28 |
| CME6_9 | 0.78 |  |  |  |  |  |  |  | 0.78 |
| CJ7_1 | 0.30 | 0.20 | 0.86 | 0.24 | 0.32 | 0.09 | 1.00 |  | 3.00 |
| CJ7_2 | 0.09 |  |  |  |  |  |  |  | 0.09 |
| CS8_1 | 0.12 |  |  |  |  |  |  |  | 0.12 |
| CS8_2 | 0.12 |  |  |  |  |  |  |  | 0.12 |
| CS8_3 | 0.40 | 0.11 | 0.01 | 0.00 | 0.18 | 0.75 | 0.36 | 0.13 | 1.95 |
| CR9_1 | 0.13 |  |  |  |  |  |  |  | 0.13 |
| CR9_2 | 0.02 | 0.22 | 0.33 | 0.46 | 0.03 |  | 0.70 | 0.00 | 1.75 |
| CR9_3 | 0.21 |  |  |  |  |  |  |  | 0.21 |
| CR9_4 | 0.02 |  |  |  |  |  |  |  | 0.02 |
| CR9_5 | 0.09 |  |  |  |  |  |  |  | 0.09 |
| CMA10_1 | 0.18 | 0.10 |  |  |  | 0.80 | 0.80 | 0.19 | 2.08 |
| CMA10_2 | 0.08 |  |  |  |  |  |  |  | 0.08 |
| CMA10_*3* | 0.38 | 0.32 |  |  | 0.25 |  |  |  | 0.95 |
| CMA10_4 | 0.18 |  |  |  |  |  |  |  | 0.18 |
| CI11_1 | 0.04 |  |  |  |  |  |  |  | 0.04 |
| CI11_2 | 0.00 |  |  |  |  |  |  |  | 0.00 |
| CI11_3 | 0.31 | 0.37 | 0.61 | 1.00 | 0.13 | 0.22 | 0.33 | 0.28 | 3.25 |
| CI11_4 | 0.95 |  |  |  |  |  |  |  | 0.95 |


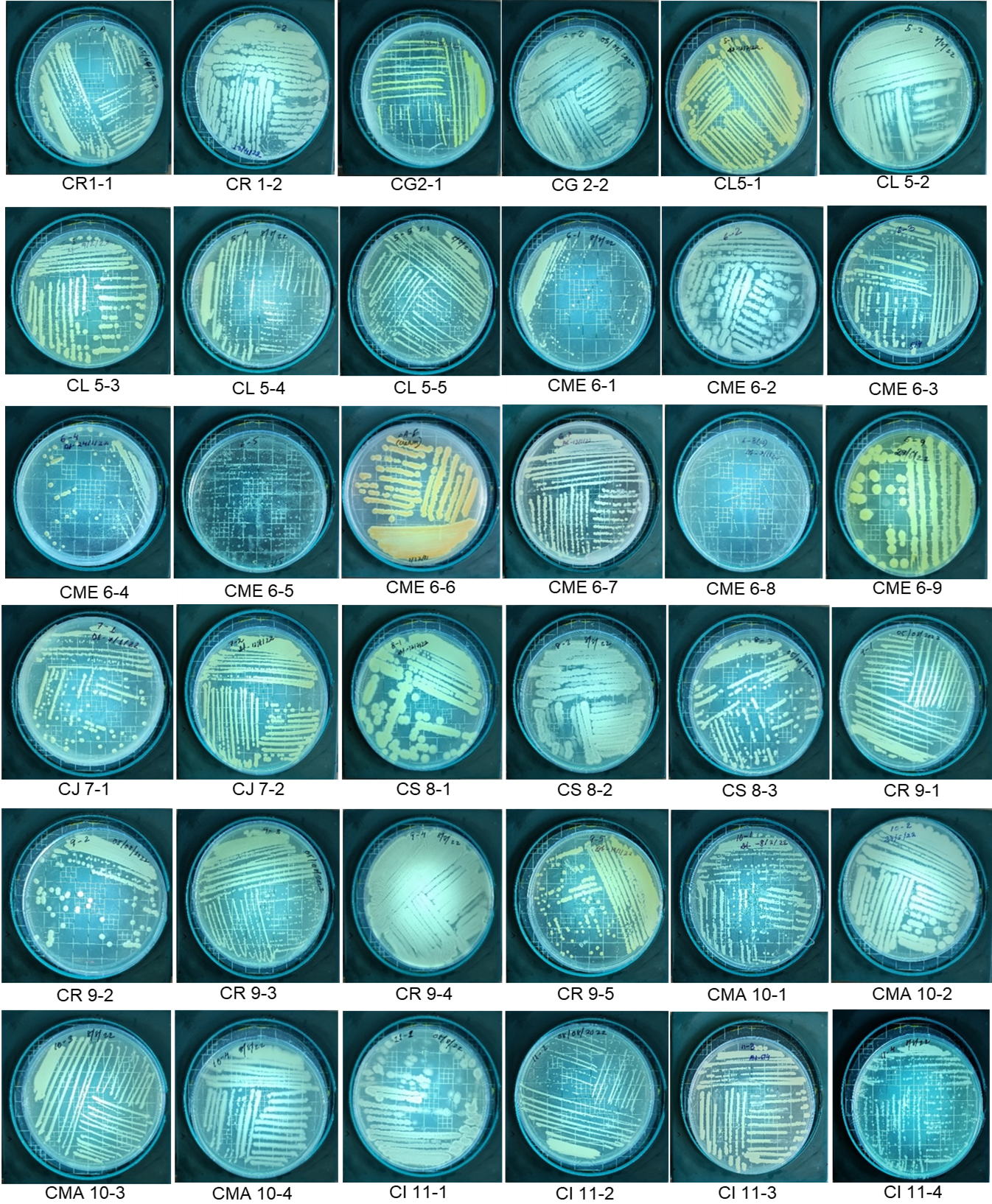


**SUPPLEMENTARY FIGURE 1. Depiction of the colony morphology of microbial isolates obtained from citrus seeds**


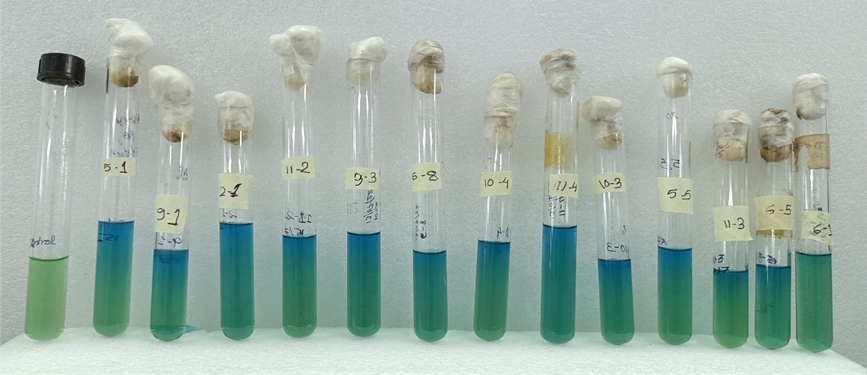


**SUPPLEMENTARY FIGURE 2. Depiction of positive results for N_2_ fixation ability in N_2_ free bromothymol blue medium**


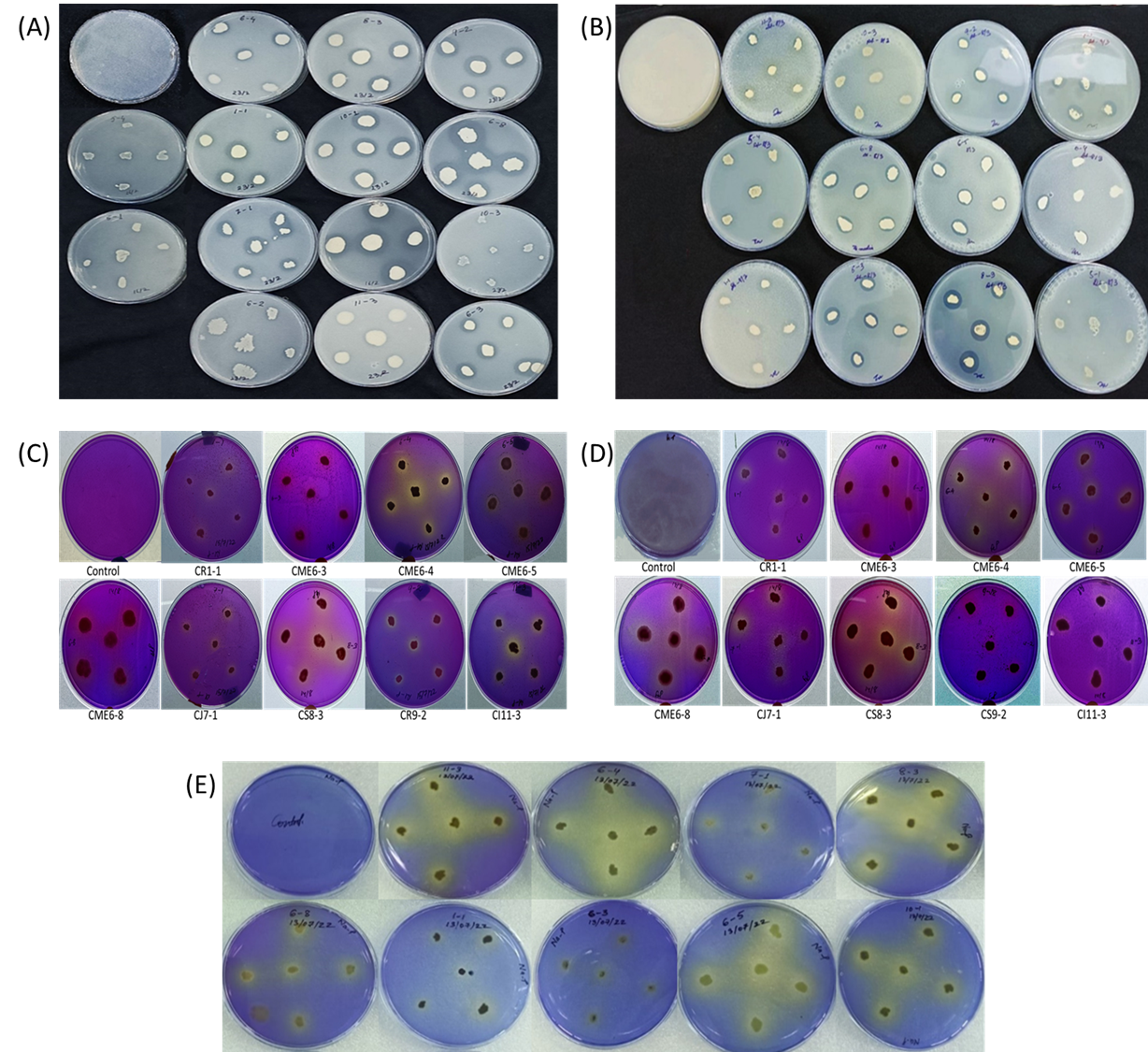
**SUPPLEMENTARY FIGURE 3. Solubilization zone formation by citrus seed isolates in Pikovskaya’s media amended with (A) Ca_3_(PO­_4_)_2_), (B) Zn_3_(PO­_4_)_2_ , (C) AlPO­_4_, (D) FePO­_4_ & (E) Na-Phytate**


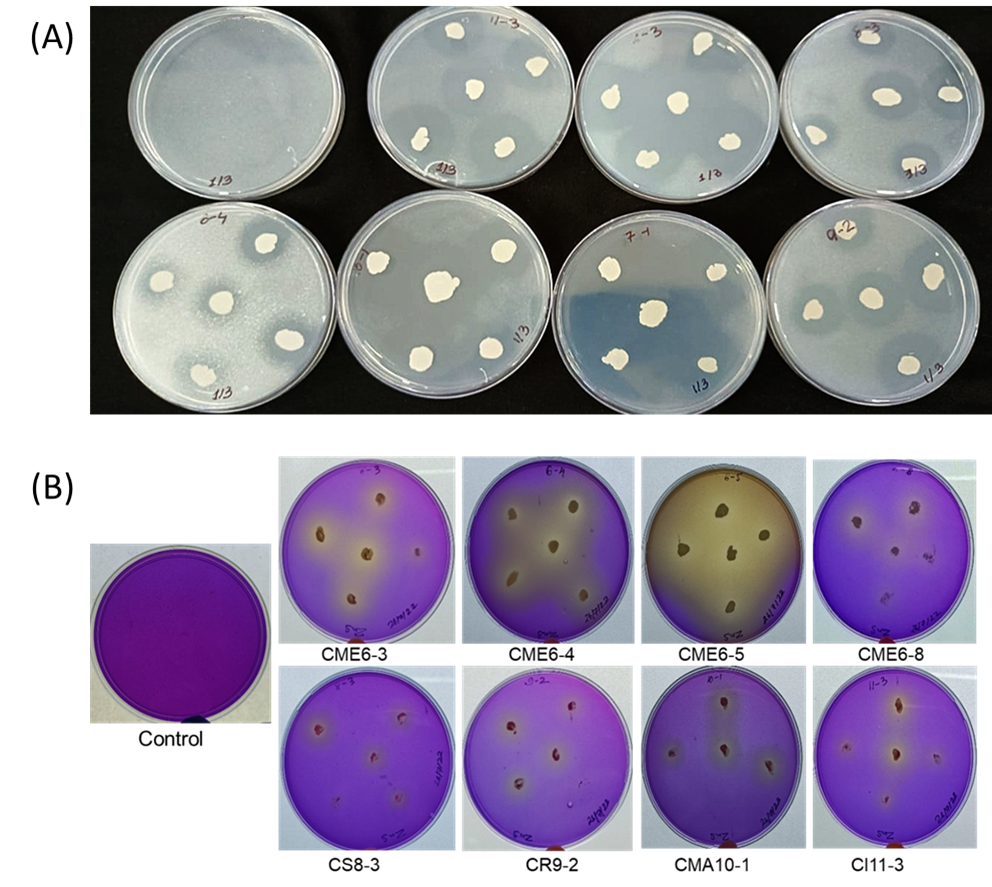


**SUPPLEMENTARY FIGURE 4. Zinc solubilization zone in Bunt and Rovira** **media amended with (A) 0.1% ZnO, (B) 0.1%ZnSO_4_**
